# Baseline Oxidative Stress Is Associated with Memory Changes in Omega-3 Fatty Acid Treated Coronary Artery Disease Patients

**DOI:** 10.1155/2017/3674371

**Published:** 2017-11-02

**Authors:** Graham Mazereeuw, Nathan Herrmann, Ana C. Andreazza, Gustavo Scola, David W. L. Ma, Paul I. Oh, Krista L. Lanctôt

**Affiliations:** ^1^MD Program, University of Toronto, Toronto, ON, Canada; ^2^Hurvitz Brain Sciences Program, Sunnybrook Research Institute and Department of Psychiatry, University of Toronto, Toronto, ON, Canada; ^3^Centre for Addiction and Mental Health and Departments of Psychiatry and Pharmacology/Toxicology, University of Toronto, Toronto, ON, Canada; ^4^Centre for Addiction and Mental Health and Department of Psychiatry, University of Toronto, Toronto, ON, Canada; ^5^Department of Human Health and Nutritional Sciences, University of Guelph, Guelph, ON, Canada; ^6^University Health Network at Toronto Rehabilitation Institute, Toronto, ON, Canada; ^7^Hurvitz Brain Sciences Program, Sunnybrook Research Institute and Departments of Psychiatry and Pharmacology/Toxicology, University of Toronto, Toronto, ON, Canada

## Abstract

**Objective:**

This study investigated whether pretreatment oxidative stress, measured by lipid hydroperoxides (LPH), 4-hydroxy-2-nonenal (4-HNE), 8-isoprostane (8-ISO), and malondialdehyde (MDA), was associated with improvement in immediate recall among n-3 PUFA-treated coronary artery disease patients.

**Methods:**

This was a secondary analysis of the CAROTID trial (NCT00981383). Composite immediate recall, measured using the California Verbal Learning Test, Second Edition, and the Brief Visuospatial Memory Test-Revised, was assessed. LPH, 4-HNE, 8-ISO, MDA, and n-3 PUFA concentrations were analysed from fasting blood. Patients then received either n-3 PUFA treatment or placebo for 12 weeks, after which composite immediate recall was reassessed. Linear regression was used to investigate relationships between lipid peroxidation markers and changes in composite immediate recall in each treatment group.

**Results:**

Eighty-five patients (age = 61.1 ± 8.5, 77% male, mean years of education = 15.3 ± 3.4) were included (*n* = 46 placebo, *n* = 39 n-3 PUFA). After adjusting for multiple comparisons and potential confounders, greater baseline concentrations of LPH (*β* = 0.45, *p* = .002) and 4-HNE (*β* = 0.38, *p* = .005) were associated with greater improvement in composite immediate recall among n-3 PUFA-treated patients. No other associations were observed.

**Conclusions:**

N-3 PUFA treatment may be more likely to improve immediate recall in patients with greater oxidative stress.

## 1. Introduction

Patients with coronary artery disease* (CAD)* more commonly demonstrate subtle cognitive deficits [1, 2] and are at increased risk for dementia relative to those without CAD [3, 4]. Decline in immediate recall appears to precede multidomain cognitive decline as both cognitively normal individuals and patients with mild cognitive impairment progress toward dementia [5, 6]. Accordingly, deficits in immediate recall may be important to monitor and remediate in patients with CAD.

As no preventative treatments for dementia currently exist, the potential procognitive effects of omega-3 polyunsaturated fatty acid* (n-3 PUFA)* supplementation have been investigated by a multitude of clinical trials [7, 8], including a recent trial in CAD patients [9]. Results of those trials indicate that although n-3 PUFA treatment is generally inefficacious for improving cognition, immediate recall may be a treatment-responsive domain [7]. However, immediate recall response to n-3 PUFA treatment is heterogeneous and this heterogeneity is likely unrelated to study-level differences [7]. These findings suggest that pathophysiological differences between patients may be a potential factor in treatment-response variability.

Oxidative stress is a central component of CAD pathophysiology [10] and has been associated with both cognitive deficits and decline [11–13]. N-3 PUFA has demonstrated antioxidant effects in clinical samples [14, 15], and those effects have been related to improvements in memory in animal studies [16]. We hypothesized that n-3 PUFA treatment efficacy for immediate recall might be greater among patients with greater oxidative stress prior to treatment.

This study investigated whether baseline concentrations of the oxidative stress markers lipid hydroperoxides* (LPH)*, 4-hydroxy-2-nonenal* (4-HNE)*, 8-isoprostane* (8-ISO)*, and malondialdehyde* (MDA)* were associated with improvement in immediate recall among n-3 PUFA-treated CAD patients.

## 2. Methods

This was a secondary analysis of the cognitive outcomes from the CAD Randomized Omega-3 Trial in Depression* (CAROTID)*, a 12-week, parallel-arm trial of 1.9 g/day n-3 PUFA treatment (including 1.2 g/day eicosapentaenoic acid* (EPA)* and 0.6 g/day docosahexaenoic acid* (DHA)*) in CAD patients (NCT00981383) [9]. This study was approved by the Research Ethics Boards of Sunnybrook Health Sciences Centre, University Health Network, and Trillium Health Partners and was conducted according to the principles expressed in the Declaration of Helsinki.

### 2.1. Patients

Trial inclusion and exclusion criteria are detailed elsewhere [9]. Briefly, patients enrolled in CAROTID were those with evidence of stable CAD (history of myocardial infarction, coronary artery bypass graft, percutaneous transluminal coronary angioplasty, or at least a 50% stenosis in one or more major coronary artery), aged 45–80 years, male or female, and with the ability to speak and understand English. All eligible patients, with or without depression, were included. Excluded patients were those with a significant acute medical illness, clinically significant cognitive impairment (Standardized Mini-Mental State Examination score < 24 or a diagnosis of dementia), a neurological condition, unstable angina, or a contraindication to n-3 PUFA supplement use. Antidepressant use was permitted if used at a stable dose for at least 3 months prior to the trial.

### 2.2. Design

Eligible patients were invited to a prerandomization baseline visit, at which, demographic, anthropomorphic, medical, and medication information was documented. Immediate recall performance was assessed using the immediate recall components of the California Verbal Learning Test, Second Edition [17], and the Brief Visuospatial Memory Test-Revised [18]. Raw scores from each test were adjusted for population norms and the resulting* Z*-scores were used in the analysis. Composite immediate recall was the primary outcome measure and was calculated by the mean of verbal and visuospatial recall* Z*-scores for each patient. Depressive symptom severity was measured using the 17-Item Hamilton Depression Rating Scale* (HAM-D)* [19] and was accounted for as a covariate. Fasting (12 hours overnight) blood was drawn and processed for analysis of serum lipid peroxidation markers and plasma n-3 PUFAs, which were a planned covariate. Patients were then randomized (1 : 1) to receive either 1.9 g/day n-3 PUFA supplements or placebo for 12 weeks. Composite immediate recall performance was reassessed after 12 weeks.

### 2.3. Analysis of Lipid Peroxidation Markers and n-3 PUFA

All samples for each lipid peroxidation marker were analysed in the same batch to eliminate batch-to-batch variability. LPH was measured using a colorimetric LPH assay kit with slight modifications (Cayman Chemical, item number 705002). LPH radicals were extracted from serum into chloroform and then mixed with the LPH assay kit. This kit measured the LPH radicals directly through redox reactions with ferrous ions, which were detected using thiocyanate as the chromogen. Each sample was plated in triplicate and the average serum LPH concentration for each sample was determined by converting the resulting absorbance (500 nm) of each sample in spectrophotometry to *μ*mol/L using a hydroperoxide concentration standard. The sensitivity of our technique is between 0.5 and 45.00 *μ*mol/L of hydroperoxides. The interassay coefficient of variation was between 0.0% and 17.2%.

4-HNE protein adducts, via Michael addition to lysine, histidine, or cysteine, were measured using ELISA (STA-838; Cell Biolabs). 4-HNE in serum was mixed with bovine serum albumin, incubated overnight, and then mixed with anti-HNE polyclonal antibody and a horseradish peroxidase conjugated secondary antibody. Each sample was plated in triplicate and the average 4-HNE concentration for each sample was determined by converting the resulting absorbance of the horseradish peroxidase reaction with a substrate solution in spectrophotometry (450 nm) to fmol/*μ*g using a protein standard. The sensitivity of this assay is between 3.9 and 250 fmol/*μ*g of 4-HNE-bovine serum albumin. The interassay coefficient of variation was between 0.0% and 25.9%, with 95.3% (81/85) of samples yielding an interassay coefficient of variation lower than 25.0%.

8-ISO was measured using a standard competitive sandwich ELISA (#516351; Cayman Chemical) according to manufacturer's instructions. Serum 8-ISO was conjugated to acetylcholinesterase, with the complex then bound to rabbit IgG mouse monoclonal antibody in the well of the ELISA plate. Each sample was plated in duplicate and the average 8-ISO concentration for each sample was determined by converting the resulting absorbance of acetylcholinesterase reaction with a substrate solution in spectrophotometry (412 nm) to pg/mL using a protein standard. The sensitivity of this assay is between 0.8 and 500 pg/mL. The interassay coefficient of variation was between 0.0% and 28.7%, with 98.8% (84/85) of samples yielding an interassay coefficient of variation lower than 25.0%.

Serum MDA concentrations (Cayman; item number 700870) were measured based on the absorbance of thiobarbituric acid reactive substances in spectrophotometry (540 nm). MDA-thiobarbituric acid adducts were generated in acidic and high temperature (90–100°C) conditions. Each sample was plated in duplicate and the average MDA concentration for each sample was determined by converting the resulting absorbance to *μ*mol/L using a thiobarbituric acid standard. The sensitivity of this assay is between 0 and 50 *μ*mol/L. The interassay coefficient of variation was between 0.0% and 22.6%.

Plasma concentrations of EPA, DHA, and the n-6 PUFA, arachidonic acid* (AA)*, were measured by gas chromatography as previously described [20]. All analyses were performed blinded to treatment allocation and patient characteristics.

### 2.4. Statistical Analyses

Data missing due to dropout were imputed using the multiple imputation method [21], and the resulting dataset was used for the primary analysis. Baseline concentrations of LPH, 4-HNE, 8-ISO, and MDA were each assessed as predictors of change in composite immediate recall* Z*-scores over 12 weeks in both the n-3 PUFA and placebo groups using linear regression. Each model consisted of change in the composite immediate recall* Z*-score as the dependent variable, with baseline composite immediate recall* Z*-score and the lipid peroxidation marker being studied as independent variables. Results for each marker in each treatment group were adjusted for the false discovery rate [22], and only those remaining significant were investigated further.

The ratio of baseline EPA and DHA concentrations to AA concentrations* (EPA + DHA/AA)* was calculated and included as a planned covariate given the relationships of those fatty acids with cognitive performance [23, 24]. Baseline HAM-D score was an additional planned covariate as depressive symptoms may also influence cognitive performance [9].

In post hoc analyses, observed relationships between baseline lipid peroxidation marker concentrations and changes in composite immediate recall* Z*-scores were assessed in the per-protocol subgroup of patients. Additionally, composite immediate recall* Z*-scores were deconstructed into verbal and visuospatial recall* Z*-scores, and relationships with lipid peroxidation markers were explored in each domain.

Statistical models were computed using SPSS statistical software, version 13.0, Chicago, IL, USA, and all analyses were two-tailed.

## 3. Results

As detailed elsewhere [9], 92 patients with CAD were enrolled in CAROTID. Despite adequate treatment compliance, patients using n-3 PUFA did not demonstrate improvement in immediate recall over 12 weeks relative to those using placebo. Of those randomized, 85 patients provided baseline serum lipid peroxidation samples and were included in this study.

Patients in each treatment group were similar (Table 1) with respect to age, sex, years of education, cardiovascular history, medication use, baseline immediate recall performance, baseline EPA + DHA/AA ratio, and baseline concentrations of the lipid peroxidation markers. Mean depressive symptom severity was mild and was similar between groups.

**Figure 1:**
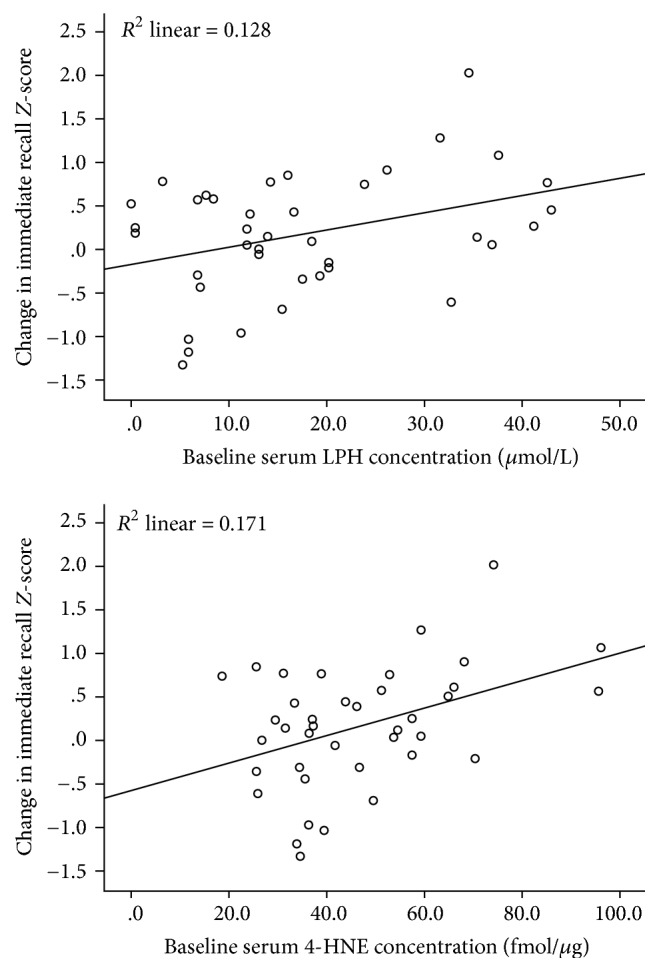
Associations of baseline LPH and 4-HNE concentrations with changes in composite immediate recall* Z*-scores over 12 weeks of n-3 PUFA treatment (*n* = 39).

Greater baseline concentrations of LPH and 4-HNE were significantly associated with greater improvement in composite immediate recall among patients receiving 12 weeks of n-3 PUFA treatment (Table 2, Figure 1), after correcting for multiple comparisons. Baseline concentrations of 8-ISO and MDA were not associated with changes in composite immediate recall in n-3 PUFA-treated patients. Similarly, there were no associations between baseline LPH, 4-HNE, or 8-ISO and changes in composite immediate recall among patients using placebo. A significant relationship between greater baseline MDA concentrations and decline in composite immediate recall over 12 weeks was observed in the placebo group; however, it did not survive correction for the false discovery rate.

Greater baseline concentrations of LPH (B (SE) = 0.02 (0.01), *β* = 0.39, *p* = .013), and 4-HNE (B (SE) = 0.01 (0.01), *β* = 0.29, *p* = .047) remained associated with greater improvement in composite immediate recall over 12 weeks of n-3 PUFA treatment after adjusting for baseline EPA + DHA/AA ratios and baseline HAM-D scores as covariates.

### 3.1. Post Hoc Analyses

In the per-protocol subgroup of patients treated with n-3 PUFAs (*n* = 34), greater baseline concentrations of LPH (B (SE) = 0.03 (0.01), *β* = 0.47, *p* = .002) and 4-HNE (B (SE) = 0.01 (0.01), *β* = 0.39, *p* = .013) remained significantly associated with greater improvement in composite immediate recall with 12 weeks of n-3 PUFA treatment.

Deconstructing composite immediate recall into verbal recall and visuospatial recall revealed that greater baseline concentrations of LPH (verbal recall: B (SE) = 0.01 (0.01), *β* = 0.18, *p* = .31; visuospatial recall: B (SE) = 0.04 (0.01), *β* = 0.46, *p* = .001) and 4-HNE (verbal recall: B (SE) = 0.01 (0.01), *β* = 0.20, *p* = .17; visuospatial recall: B (SE) = 0.02 (0.01), *β* = 0.34, *p* = .014) were particularly associated with improvement in visuospatial recall after n-3 PUFA treatment.

## 4. Discussion

This secondary analysis of cognitive outcomes from the CAROTID trial found that higher baseline concentrations of the lipid peroxidation markers LPH and 4-HNE, indicating greater pretreatment levels of oxidative stress, were associated with greater improvements in immediate recall in CAD patients after 12 weeks of n-3 PUFA treatment. The fact that no such associations were observed among patients using placebo and that known predictors of cognitive change, such as age, years of education, and cardiovascular risk factors [25], were balanced at baseline between the treatment groups accords with a potential relationship between oxidative stress and n-3 PUFA treatment efficacy.

To our knowledge, this is the first study to examine the relationship between baseline markers of oxidative stress and n-3 PUFA efficacy on memory or other cognitive domains. However, markers of oxidative stress and inflammation have been previously associated with n-3 PUFA treatment response in other conditions, such as depression [26, 27], which aligns with our hypothesis that oxidative stress may be relevant to the success of n-3 PUFA supplementation and may underlie its variable efficacy in previous trials [7–9].

Mechanistically, n-3 PUFAs appear to have antioxidant effects [28]. In particular, EPA and DHA have been shown to reduce the production of hydroxyl and superoxide radicals, in turn reducing the production of downstream reactive oxygen species that can attack lipids and proteins. Such antioxidant effects have been observed in both animal studies [29–32] and clinical studies involving patients with mild cognitive deficits [15]. Furthermore, animal studies have shown that n-3 PUFA antioxidant effects correlate with improved memory performance [16, 33, 34]. It is unclear why LPH and 4-HNE were the only markers associated with n-3 PUFA treatment benefits. Preclinical studies have not clearly revealed the conditions under which LPH may differentially convert to 4-HNE, MDA, or 8-ISO* in vivo* and so we cannot speculate on the reason for our findings in this clinical sample. However, all four markers have been linked with memory deficits [35–38], as well as n-3 PUFA antioxidant effects [29–32] in animal studies, suggesting the potential for their involvement. Replication of our study may clarify which markers are most relevant to n-3 PUFA treatment effects on memory.

### 4.1. Strengths and Limitations

A strength of this study is that important predictors of cognitive change such as age, years of education, and cardiovascular risk factors were balanced between the treatment groups and were therefore not required as covariates. As such, we could examine the relationship between baseline oxidative stress and cognitive change in each treatment group using a relatively small sample size. However, the small size precluded examination of that relationship in a more powerful interaction (oxidative stress × treatment × time) model combining both treatment groups. Another strength was our ability to account for depressive symptoms, which have been previously shown to influence cognitive performance [9, 39] and are highly prevalent in the CAD population [40].

A limitation of this study was that the included patients, despite demonstrating a range of baseline immediate recall performance, were all cognitively healthy and may therefore have been limited in their potential for cognitive change. However, the measures of immediate recall used in this study both provide a high ceiling for potential scores and none of the included patients approached the maximum at either study visit. Moreover, such a limitation would apply equally to both treatment groups and is unlikely to have confounded our findings. How baseline oxidative stress may be associated with cognitive changes among patients with greater cognitive deficits remains to be investigated. Finally, our findings are limited to the domain of immediate recall and to the CAD population. As such, they are not necessarily generalizable to cognitively healthy individuals without cardiovascular disease or patients with cognitive deficits secondary to other vascular diseases or nonvascular causes.

## 5. Conclusions

After adjusting for multiple comparisons and potential confounders, greater baseline concentrations of LPH and 4-HNE were associated with greater improvement in immediate recall performance among CAD patients after 12 weeks of n-3 PUFA treatment. Our finding may clarify the variability in cognitive response to n-3 PUFA observed in previous trials. Future research is warranted to assess whether these markers have clinical utility.

## Figures and Tables

**Table 1:** Baseline participant characteristics.

Variable, mean (SE)	Placebo (*n* = 46)	N-3 PUFA (*n* = 39)	*F*/*χ*^2^	*p* value
Age	59.8 (1.2)	62.6 (1.4)	2.13	.15
Male, %	74	80	0.44	.51
Education, yrs	15.7 (0.5)	14.9 (0.6)	1.17	.28
*Cardiovascular history*				
Event/procedure^*∗*^			0.80	.85
MI, %	39	31		
PTCA, %	37	38		
CABG, %	22	28		
Vascular risk factors, # of 5^*∗∗*^	2.8 (0.2)	3.1 (0.2)	0.89	.35
VO_2_ peak, % age and sex norm	73% (24%)	73% (20%)	0.03	.86
*Medications*				
Antidiabetic, %	13	26	2.17	.14
Antihypertensive, %	69	82	1.86	.17
Anti-inflammatory, %	4	2	0.22	.64
Platelet inhibitor, %	98	92	1.43	.21
Statin, %	100	98	1.16	.28
*Psychometric performance*				
Composite recall, *Z*-score	0.23 (0.16)	0.06 (0.16)	0.61	.44
Verbal recall, *Z*-score	0.37 (0.16)	0.30 (0.16)	0.10	.75
Visuospatial recall, Z-score	0.08 (0.19)	−0.19 (0.18)	1.02	.32
HAM-D score	7.6 (0.8)	6.8 (1.1)	0.32	.57
*Blood markers*				
Plasma EPA + DHA/AA ratio	0.34 (0.03)	0.33 (0.02)	0.12	.73
Serum LPH, *μ*mol/L	18.2 (1.9)	17.7 (2.0)	0.03	.87
Serum 4-HNE, fmol/*μ*g	46.4 (2.2)	44.5 (2.7)	0.27	.60
Serum 8-ISO, pg/mL	0.13 (0.01)	0.11 (0.01)	1.06	.31
Serum MDA, *μ*mol/L	0.04 (<0.01)	0.04 (<0.01)	0.02	.89

SE, standard error; N-3 PUFA, n-3 polyunsaturated fatty acid; *F*, *F*-statistic of analysis of variance; *χ*^2^, chi-squared test statistic; MI, myocardial infarction; PTCA, percutaneous transluminal coronary angioplasty; CABG, coronary artery bypass graft; VO_2_ peak, peak volume of oxygen uptake during cardiac stress test; HAM-D, Hamilton Depression Rating Scale; EPA, eicosapentaenoic acid; DHA, docosahexaenoic acid; AA, arachidonic acid; LPH, lipid hydroperoxides; 4-HNE, 4-hydroxy-2-nonenal; 8-ISO, 8-isoprostane; MDA, malondialdehyde. ^*∗*^Patients may have had both an event and one or more procedures. ^*∗∗*^Vascular risk factors: hypertension, obesity (body mass index ≥ 30), dyslipidemia, diabetes mellitus, and smoking.

**Table 2:** Associations between baseline lipid peroxidation marker concentrations and changes in composite immediate recall *Z*-scores over 12 weeks by treatment group.

Outcome	Placebo (*n* = 46)			n-3 PUFA (*n* = 39)		
	B (SE)	*β*	*p* value	B (SE)	*β*	*p* value
LPH	0.01 (0.01)	0.05	.70	0.25 (0.01)	0.45	*.002* ^*∗*^
4-HNE	−0.01 (0.01)	−0.07	.59	0.14 (0.01)	0.38	*.005* ^*∗*^
8-ISO	−1.07 (1.26)	−0.12	.39	−0.65 (2.00)	−0.05	.75
MDA	−12.40 (5.18)	−0.32	.017	−4.28 (4.70)	−0.14	.36

*Note*. B and *β* are the unstandardized and standardized regression coefficients, respectively. SE is the standard error of the B coefficient. *∗* Indicates that the result remained statistically significant after adjustment for false discovery rate (first threshold at <.0125, second threshold at <.025; Benjamini & Hochberg). Significant *p* values are shown to three decimal places for clarity.
